# Linking transformations of organic carbon to post-treatment performance in a biological water recycling system

**DOI:** 10.1016/j.scitotenv.2020.137489

**Published:** 2020-06-15

**Authors:** Christopher Ziemba, Odile Larivé, Eva Reynaert, Theo Huisman, Eberhard Morgenroth

**Affiliations:** a Eawag: Swiss Federal Institute of Aquatic Science and Technology, 8600 Dübendorf, Switzerland; b ETH Zürich, Institute of Environmental Engineering, 8093 Zürich, Switzerland

**Keywords:** AOC, assimilable organic carbon, BAMBi, biologically activated membrane bioreactor, LC-OCD, liquid chromatography – organic carbon detector, Biologically activated membrane bioreactor (BAMBi), Decentralized, Greywater, Electrolysis, Granular activated carbon (GAC)

## Abstract

Ozone, electrolysis and granular activated carbon (GAC) were examined as potential post-treatments to follow a household-scale biologically activated membrane bioreactor (BAMBi), treating a wash water containing trace urine and feces contamination. Each post-treatment was evaluated for abilities and reaction preferences to remove or transform dissolved organic carbon (DOC), chemical structures that contribute color, and assimilable organic carbon (AOC), which can support bacterial regrowth. Batch treatment with each technology demonstrated an ability to remove ≥95% DOC. Ozone demonstrated a reaction selectivity through increased reaction rates with larger compounds and color-contributing compounds. Electrolysis and GAC demonstrated generally less-selective reactivity. Adding post-treatments to full-scale systems reduced DOC (55–91%), AOC (34–62%), and color (75–98%), without significant reaction selectivity. These reductions in DOC and AOC were not linked to reduction of bacterial concentrations in treated water. Reductions in bacterial concentrations were observed with ozone and electrolysis, but this is credited to oxidation chemicals produced in these systems and not the removal or transformations of organic materials.

## Highlights

• Ozone, electrolysis and GAC can effectively remove DOC and color in batch testing.
• Ozone is selective (others are not) to remove larger and color-contributing organics.
• No differential carbon selectivities were observed between treatments at full-scale.
• Anti-microbial chemicals are needed to combat regrowth. AOC was not low enough.
• A BAMBi-GAC-electrolysis system is a promising solution for greywater recycling.

## Graphical abstract

**Figure:**
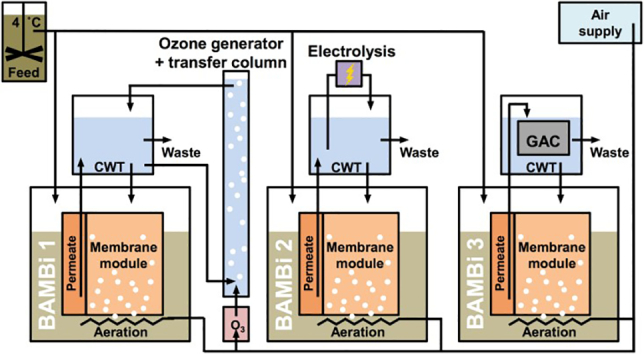

## 1 Introduction

Recycling greywater or other wastewaters for high-quality applications, such as hand washing, can dramatically reduce global demand for freshwater and enable safer water access for improved hygiene in the developing world and other challenging environments. This opportunity has inspired a new wave of research and development into advanced greywater and blackwater recycling systems that employ advanced process technologies to achieve improved water quality beyond what has traditionally been required for irrigation or toilet flushing (Gassie and Englehardt, 2017; Nguyen et al., 2017; Dubowski et al., 2019; Ziemba et al., 2019). This growing interest in wastewater reuse has been accompanied by the development and implementation of guidelines and standards that regulate the composition of the treated water. Most recently, the International Organization for Standardization (ISO) has published a standard for non-sewered sanitation systems (ISO 30500), including specific requirements for effluent quality and biological safety (ISO, 2018). Additionally, to the requirements stated in ISO 30500, the requirements of the WHO Guidelines for Drinking Water Quality must be met if the treated water is intended to be used for hand washing. At the most fundamental level, recycled hand washing water must not contain undesirable color or odor and must not support harmful growth of pathogens (WHO, 2017). Color, odor and pathogen growth concerns are each linked to the concentrations and chemical structure of the organic carbon content of the water, and excessive or problematic carbon must be removed or transformed during treatment. For existing greywater systems that have demonstrated promising performance and economic feasibility, it may be beneficial to add a post-treatment step to achieve treatment objectives for carbon and other objectives.

One proven system for on-site water recycling of low-strength wastewater is a biologically activated membrane bioreactor (BAMBi) (Künzle et al., 2015; Ravndal et al., 2015). This system was designed to provide onsite recycling of wash water in urban slums at the household level (Supporting information S.8 and Tobias et al., 2017) and employs many of the recognized advantages (permanent barrier, economic feasibility, reduced footprint) of membrane-based systems (Wu, 2019). Previous field testing of this gravity driven membrane treatment system has demonstrated a 95% removal of organic carbon during the repeated recycling of a wash water comprised of greywater from handwashing and anal cleansing water (Künzle et al., 2015). Unfortunately, the produced water still contained significant dissolved organic carbon concentrations (35–40 mg/L) that contributed to a yellow color (Künzle et al., 2015) and can fuel the growth of pathogens (Nguyen et al., 2017). Safe and effective application of BAMBi and similar biological membrane treatment systems to treat similar wastewaters to a hand washing quality requires supplemental treatment.

While many technologies have demonstrated effective performance in a diversity of drinking water and wastewater applications, there exists a specific knowledge gap in understanding and selecting post-treatment technologies to achieve aesthetic and microbial safety targets in decentralized water recycling applications. This knowledge gap exists because, (i) the performance of a specific technology can strongly depend on the specific water chemistry and environmental conditions of any given application, and (ii) decentralized water recycling for higher-quality applications (including human contact) is not a well-developed field of research. This project considered ozone, electrolysis and granular activated carbon (GAC) as potential post-treatment technologies because ozone and GAC are well-understood and broadly-applied technologies in the drinking water and wastewater fields, and because electrolysis offers the potential to produce oxidation species more powerful than ozone.

Ozone can effectively remove taste, odor, and color (Camel and Bermond, 1998). The reactivity of ozone (2.07 V oxidation potential) is highly selective to oxidize electron-rich moieties such as double or triple bonds, aromatic structures and non-protonated amines (Hoigné and Bader, 1983; Von Gunten, 2003). The implication of adding ozone to a system can be highly dependent on the specific chemistry of that system. Ozone is itself capable of inactivating bacteria, including those resistant to chlorination (Ding et al., 2019), but it has also been demonstrated to produce AOC, which supports bacterial growth (van der Kooij et al., 1989; Chen et al., 2018).

Electrolysis is a well-established electrochemical oxidation technology for removal of organic pollutants and color from wastewaters (Martínez-Huitle et al., 2015). Effective oxidation through electrolysis generally depends on maximizing the production of highly-oxidative compounds such as hydroxyl radical (Martínez-Huitle et al., 2015). The higher oxidation potential of hydroxyl radical relative to ozone (2.8 vs. 2.07 V) initiates less-selective reactivity. Electrolysis treatment of wastewater can also result in the oxidation of chloride to chlorine (Martínez-Huitle et al., 2015) which is a proven antimicrobial agent.

GAC is another well-established technology for removing color, odor and recalcitrant compounds. The mechanism by which GAC removes organics can be chemical (van der Waals, dipole-dipole, and electrostatic), physical or through biological degradation conducted by bacteria on the surface of the carbon. GAC may favor smaller adsorbates because size-exclusion potentially limits pore access (Newcombe et al., 1997) and because smaller adsorbates can adsorb more strongly with increased specific interface area (Li et al., 2002). The predominantly hydrophobic character of GAC (Kilduff et al., 1996) may also promote adsorption of hydrophobic molecules, but the surface chemistry, and specifically surface charge, of the GAC may be dictated by pH (Abouleish and Wells, 2015). For pH values below the zero point charge (ZPC) of the GAC, the surface of the GAC would be positive value, and the reverse is true for pH values above the ZPC. The difference between pH and ZPC of the GAC can influence deposition, and the severity of this influence is determined by the chemical structure of the GAC.

The goal of this study is to understand how the addition of ozone, electrolysis or GAC post-treatment impacts the biologically-relevant organic carbon in a decentralized water recycling system, and also understand to what degree these impacts influence the ability of the system to deliver safe and aesthetically acceptable water. We hypothesize that the reaction selectivity of different post-treatments will indeed result in different profiles of organic materials, in terms of color, size-distribution and bio-compatibility, and that these differences will impact overall system performance. Batch testing was conducted to investigate how the reaction selectivity of each treatment specifically affects removal of DOC, AOC, color-contributing structures, and size-distributions of organic compounds. Each post-treatment was then integrated into a BAMBi system to demonstrate how any reaction selectivities impact carbon and bacterial concentrations at full-scale. The combination of targeted batch testing, and integrated full-scale performance testing provides an informed basis for designing water recycling systems that appropriately remove, transform or permit residual organic materials.

## 2 Methods and materials

### 2.1 Biologically activated membrane bioreactor (BAMBi) system

Three BAMBi systems were assembled, each consisting of a 150 kDa standing sandwich ultrafiltration membrane module (Microclear MCXL module, Newterra, Ontario, Canada) with 3 m^2^ total surface area of membrane (Microdyn-Nadir, Wiesbaden, Germany) submerged in a 50 L low-density polyethylene (LDPE) tank. The weight of the water on the feed side of the membrane pushed water through the biofilm and through the membrane into a reservoir. This gravity driven membrane operation does not require chemical cleaning or shear for the long-term maintenance of flux (Pronk et al., 2019). This permeate reservoir was pumped completely to the clean water tank (CWT) at 5 min intervals. Each BAMBi was fed 3 L/d concentrated wash water and 57 L/d of recycled water from the CWT. Wash water was modeled after the use case of on-site handwashing connected to a toilet system. Pumping water from the CWT to the BAMBi simulates the usage of the water by the user and the contribution from the concentrated feed represents the loading that the usage introduces. The concentrated feed consisted of 500 g (wet) feces, 1 L urine and 0.1 L soap added to tap water totaling 40 L, resulting in a pH of approximately 8. The soap used in this study was designed to recreate common liquid handwashing soap, while minimizing antibacterial and membrane-fouling potential. Sodium dodecyl sulfate was included as a surfactant, glycerol as a moisturizer, sodium chloride as a stabilizer and lactic acid to reduce the pH to approximately 6.5. More information regarding, BAMBi, a schematic of the BAMBi systems and a recipe for the soap are presented in Supporting information Sections S.1, S.2, and S.8.

### 2.2 Post-treatment testing

Batch testing was conducted using permeate water collected from a BAMBi system without post-treatment (pH ~ 8), filtered at 2–4 μm (640 d-cellulose, Macherey-Nagel, Düren, Germany), pasteurized at 60 °C for 1 h, cooled, filtered at 0.2 μm (polyethylsulfone 690.020.00.1, Geberit, Rapperswil-Jona, Switzerland) and stored at 4 °C. Electrolysis and ozone testing was conducted with one batch of permeate, while GAC testing was conducted with permeate collected on a different day. The permeate was then subjected to increasing exposures of ozone, electrolysis or GAC treatment until approximately 95–99% of DOC was removed, with sampling occurring at appropriate intervals.

Ozone batch testing was conducted with 6 L of permeate in a 2 m tall poly(methyl methacrylate) column. Ozone was produced by a corona-discharge ozone generator, with a maximum design output of 300 mg/h (Certizon C300, Erwin Sander Elektroapparatebau GmbH, Uetze-Eltze, Germany), fed 2 L/min compressed air, and bubbled (~4 mm diameter bubbles) into the bottom of the column using a 2.4 × 2.4 × 7.2 cm diffusion stone (Bubblemac Industries, Minneola, Florida, USA). Water was recirculated from the bottom of the column to the top at 0.5 L/min with a peristaltic pump through polytetrafluoroethylene (PTFE) tubing, and samples were collected from this tubing. Actual ozone output from the ozone generator was measured as 240 mg/h using an ozone analyzer (964 BMT Messtechnik, Berlin, Germany).

Electrolysis batch testing was conducted with 4 L of permeate in a glass bottle maintained at ~22 °C, recirculated through a boron-doped diamond electrolysis flow cell with 3 mm spacing (DiaClean® 103, WaterDiam, Delémont, Switzerland) at a rate of 0.5 L/min using PTFE tubing and a peristaltic pump. Electricity (2.5 A and 12.2 V) was supplied by a laboratory power supply with a 5 min/1 min on/off cycle, with polarity reversing between cycles. Samples were collected from the bottle.

GAC batch testing consisted of pouring 3 L of permeate water though a 6 cm diameter poly(methyl methacrylate) tube with a mesh screen holding 270 g virgin GAC. Each pass though the column corresponded to an average (empty bed) contact time of ~10 s. The GAC was 1–1.3 mm diameter in size, with a ZPC described as alkaline, derived from coal and activated by steam (Norit 830, Cabot, Boston, MA, USA).

Full-scale BAMBi testing consisted of operating three stable reactors for 30 d without post-treatment, and then 60 d with post-treatment. The same post-treatment hardware and materials were utilized as in batch testing. Water samples collected at full-scale were not subjected to the same filtration and pasteurization steps as conducted prior to batch testing. Water from the CWT was recirculated either through the ozonation column or the electrolysis cell at a flow rate of 0.5 L/min. For GAC treatment, 6 L of virgin GAC was housed in a poly(methyl methacrylate) box with a mesh bottom and inserted into the top of the CWT. The membrane permeate was pumped to the top of this filter and permitted to drain down on the way to the tank.

### 2.3 Liquid chromatography with organic carbon detector (LC-OCD) and dissolved organic carbon (DOC)

LC-OCD analysis was performed using a Toyopearl TSK HW-50S column (Tosoh Bioscience, King of Prussia, PA, USA) and an infrared detector (DOC-Labor Dr. Huber, Karlsruhe, Germany). LC-OCD data was processed using Fiffikus software (DOC-Labor Dr. Huber, version 2008-08-22) in accordance with established protocols (Huber et al., 2011), with organic compounds divided into humics, building blocks, lower-molecular weight (LMW) organics, and neutrals. The humic fraction mirrors natural humic substances. Building blocks are smaller, but have similar structure to humics, suggesting they are subunits of humics or degradation products. LMW organics are smaller still, and comprised of acids and humics. The neutrals fraction contains LMW compounds that are either weakly charged or only slightly hydrophobic. DOC was monitored using a dedicated TOC/DOC machine (TOC-L, Shimadzu, Kyoto, Japan) for full-scale testing and the LC-OCD detector for batch testing. Detailed procedures for LC-OCD and DOC protocols are presented in Supporting information Section S.3.

### 2.4 Total cell counts (TCC), intact cell counts (ICC) and assimilable organic carbon (AOC)

TCC and ICC were measured using a flow cytometer (Cytoflex, Beckman Coulter, Brea, California, USA), with SYBR® Green (Life Technologies, Eugene OR, USA) and propidium iodide (Life Technologies). AOC measurement procedure was based on an established protocol (Hammes and Egli, 2005). Detailed procedures for TCC, ICC and AOC protocols are presented in Supporting information Section S.4.

### 2.5 Chemical analysis, color and statistics

Nitrite, nitrate, chloride, sulfate and phosphate were measured by ion chromatography (Metrohm 881, Herisau, Switzerland). Ammonium was measured with gas-diffusion flow injection (Foss, Hillerød, Demark). Chlorine and ozone were measured using Dr. Lange kits (LCK 310, Hach, Loveland, Colorado, USA) employing N,N-diethyl-*p*-phenyldiamine (DPD). Color was monitored using a spectrophotometer at 400, 500 and 600 nm (Cary 60, Agilent Technologies, Santa Clara, California, USA). Only 400 nm was used in batch testing due to low signal at 500 and 600 nm. Statistical analysis was conducted using GraphPad Prism (GraphPad Software, San Diego, California, USA). Statistical analysis of full-scale systems before and after the addition of post-treatments omitted the first sampling time point after the addition of the post-treatment as a transitional period.

## 3 Results

### 3.1 Post-treatment batch testing

**Fig. 1:**
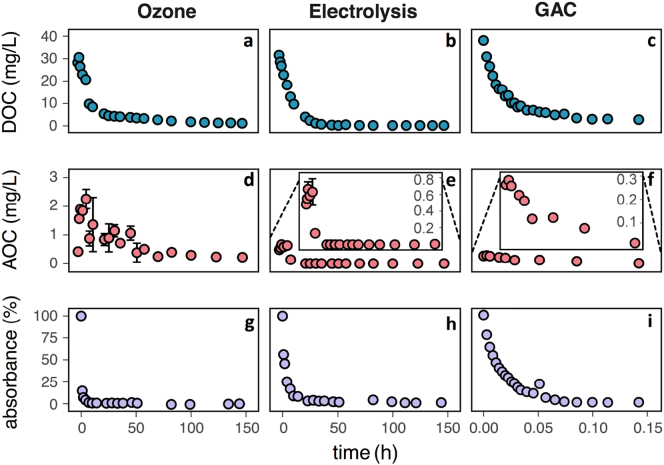
DOC, AOC and absorbance as a function of batch exposure time. Absorption is measured at 400 nm (respective to initial value). AOC values after 10 h for electrolysis are below quantification.

Each treatment effectively reduced DOC concentrations, achieving ≥95% removal (Fig. 1a–c). First-order initial DOC removal rate constants were approximately 0.1/h, 0.08/h and 59/h for ozone, electrolysis and GAC respectively. Additional samples were collected during the GAC batch experiment at increased GAC exposures. These samples have not been included in Fig. 1, but are available, along with supporting data for all batch tests, in Supporting information Section S.5.

Unlike with DOC, different post-treatments yielded different impacts on AOC (Fig. 1d–f). For ozone, the AOC concentration first increased by up to 500% of the initial concentration, then became variable about an average value of 1 mg/L between 15 and 50 h of exposure, and later reduced to a level lower than the initial value (0.2 vs. 0.4 mg/L). In electrolysis treatment, an initial increase of ~40% was observed, but then the AOC decreased to less than the quantification limit (0.002 mg/L, >99.6% removal) at 23 h exposure. The permeate water utilized in GAC testing contained a lower initial AOC concentration, but this concentration was also reduced to below the quantification level. The rate constant for the initial removal of AOC with GAC was lower than for DOC (approximate initial first-order rate constant of 15/h vs. 59/h). Overall, electrolysis and GAC post-treatments demonstrated abilities to remove both DOC and AOC (each ≥95% removal). In contrast, ozone was less effective at removing AOC (~45% removal) on the timescale on which it can remove DOC (>95% removal).

Each treatment effectively removed absorbance at 400 nm, with ultimate removals of 100% for ozone, 99% removal for electrolysis and 98% removal of GAC (Fig. 1g–i). The initial rate constant of color removal with ozone was ~3–4 times faster than the rate constant for DOC removal. Color removal was 2–3 times faster than DOC removal for electrolysis and evenly paced for GAC.

**Fig. 2:**
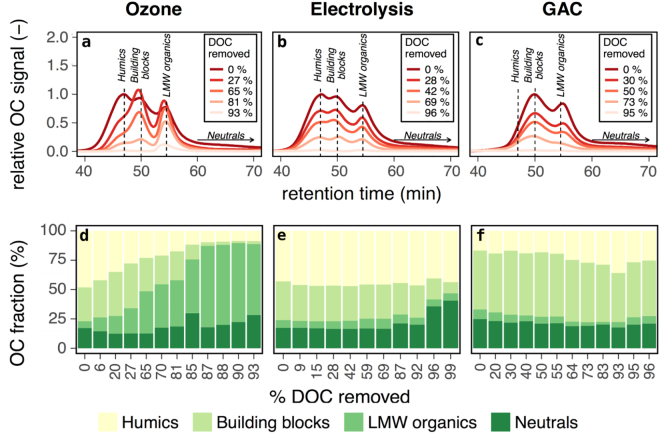
Size-distribution profiles of organic carbon (OC) from batch testing. Each sample is identified by the corresponding percentage of DOC removed by the treatment at the time of sample collection. Chromatographs in parts a-c correspond to select samples, while fraction distributions in parts d-f include additional samples.

At selected points during the batch experiments, DOC was characterized by size-distribution (Fig. 2). Ozone generally shifted the size-distribution to smaller compounds. Initially, the relative fraction of humics decreased and the relative fraction of low molecular weight (LMW) organics increased. Once the humics were removed, the relative fraction of building blocks started to decrease while the fraction of LMW organics continued to grow. Additional oxidation continued to remove larger compounds. In contrast, the size fractions of organic carbon did not change with increasing exposure to electrolysis or GAC. GAC treatment resulted in the emergence of a separate peak of compounds larger than humics. We believe that this peak does not represent a transformation of the organic molecules from the wash water. Instead we believe this peak represents a contamination of particles that break off the GAC media and enter into the water phase. This contamination peak has been excluded from the OC fraction (%) plot in Fig. 2, but is included in Supporting information Section S.5.

### 3.2 Adding post-treatments to full-scale systems

Ozone, electrolysis and GAC treatments were incorporated into full-scale BAMBi systems, with performance characterized before and after this incorporation (occurring on d 0). The overall operation of all three BAMBi units, the ozone post-treatment and the GAC post-treatment were stable during the testing duration. Unfortunately, the electricity supplied to the electrolysis was unintentionally interrupted between d 25 and 36, yet data points before and after this period present reliable data. Data collected during this interruption are thus omitted from statistical calculations.

The concentration of ozone in the CWT was consistently below the quantification limit (0.05 mg/L), with the exception of one sample. The chlorine concentrations in the CWT during electrolysis averaged ~1 mg/L-Cl_2_ free chlorine and approximately 4 mg/L-Cl_2_ total chlorine when electrolysis was working properly (Supporting information Fig. S9). Increases in ammonium (up to ~5 mg/L-N) and nitrite (up to ~3 mg/L-N) were also measured during this time. These increases may be evidence of reduction of nitrate at the cathode (Supporting information Fig. S10), but these concentrations of ammonia and free chlorine would rapidly form chloramines if they were measured at the same time in a well-mixed system. Though we do also measure chloramines, it is not entirely clear how simultaneous oxidation/reduction processes, variability in sampling times, mixing and influences of other chemicals might have impacted these measurements.

**Fig. 3:**
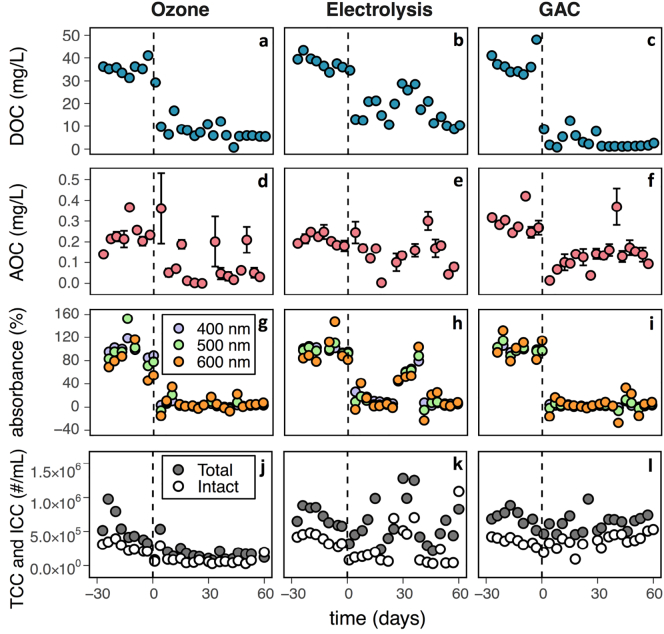
Clean water tank before and after addition (on d 0) of post-treatments. Absorbance values are presented as a percentage of the mean absorbance values before implementing the post-treatments. Due to an operation error, no electricity was provided for the electrolysis between d 25 and 36. A color version of this figure is available in the online version of this article (changes in absorbance at 400, 500 and 600 nm are similar for each wavelength). CWT: clean water tank.

Average DOC in the CWTs reduced with the addition of each post-treatment, 79% reduction for ozone, 58% reduction for electrolysis and 91% reduction for GAC (Fig. 3a–c). Each change was statistically significant (p < 0.001, 1-tailed *t*-test). These reductions represent a combination of improvement in the water quality of the permeate produced by the BAMBi (Supporting information Figs. S12–S14) and DOC removal achieved each time the water was exposed to the post-treatment. The steady-state differences in DOC between the permeate and the CWT, which can be attributed to the post-treatments, were 3.0 mg/L for ozone, 4.6 mg/L for electrolysis and 4.5 mg/L for GAC. In comparison, >55 mg/L removal was attributed to the BAMBi. Average AOC in the CWT reduced 62% (to 0.09 ± 0.1 mg/L) with ozone addition, 30% (to 0.15 ± 0.09 mg/L) with electrolysis addition, and 55% (to 0.13 ± 0.08 mg/L) with GAC addition. Ozone and GAC produced statistically significant differences (p < 0.001, 1-tailed *t*-test), while the electrolysis treatment did not (p = 0.06, 1-tailed *t*-test). Addition of all three post-treatments demonstrated significant (p < 0.001, 1-tailed *t*-test) reductions in absorbance in the CWT water (Fig. 3g–i). Absorbance at 400 nm decreased 97% for ozone, 85% for electrolysis and 98% for GAC. Similar improvements were observed for longer wavelengths. All post-treatments produced water that looked clear to the human eye (corresponding to a reduction in absorbance ≥85% at 400 nm). When comparing the relative removals of DOC, AOC and color, all three treatments demonstrated greater reductions in color than DOC, and greater reductions in DOC than AOC. The size-distributions of organic materials in the water did not change following the incorporation of ozone, electrolysis or GAC post-treatments (Supporting information Fig. S15).

TCC and ICC in the clean water tank during full-scale testing are presented in Fig. 3j–l. Ozone post-treatment decreased average TCC by 69% (to 1.7 × 10^5^ cells/mL) and average ICC by 70% (to 8.4 × 10^4^ cells/mL). The TCC achieved with ozone is in the same order of magnitude as that of drinking water in Zürich, Switzerland (8.97 × 10^4^ cells/mL) (Hammes et al., 2010). Electrolysis decreased average TCC by 31% (to 5.0 × 10^5^ cells/mL) and average ICC by 47% (to 2.1 × 10^5^ cells/mL). GAC post-treatment was less effective, with average TCC values decreasing by 13% (to 6.1 × 10^5^ cells/mL) and average ICC values decreasing by only 1%. ICC is a measure of viability, while TCC is only a measure of presence. Our results indicate that addition of ozone, electrolysis or GAC treatments does not strongly impact the ICC/TCC ratio.

### 3.3 Investment and operating costs

Investment and operating costs are presented in Table 1. The information is a combination of manufacturer information and informed estimates, described in detail in Supporting information Section S.7. With 1.56 USD/d, electrolysis is the most expensive post-treatment in terms of both investment costs and operation costs. Costs of ozone post-treatment are approximately one third of this value due to considerably lower investment costs. GAC is inexpensive and does not require any additional energy and has, for these reasons, the lowest cost of all three post-treatments (0.02 USD/d).

**Table 1:** Investment and operating costs for ozone, electrolysis and GAC in US Dollars (USD).

Equipment	Ozone	Electrolysis	GAC	Formula
Core technology				
Price (USD)	350	1100	6	
Lifespan (d)	1095	800	365	
Power (W)	10	30.5		
ON time (h/d)	24	20		
Water recirculation pump				
Price (USD)	12	12		
Lifespan (d)	1095	1095		
Power (W)	3.5	3.5		
ON time (h/d)	24	24		
Aeration pump				
Price (USD)	30			
Lifespan (d)	1095			
Power (W)	3.5			
ON time (h/d)	24			
Capital cost (USD/d)	0.36	1.39	0.02	∑*Price*_*i*_/*Lifespan*_*i*_
Avg. power (Wh/d)	408	694	0	∑*Power*_*i*_ ∙ *ON time*_*i*_
Energy costs (USD/d)	0.1	0.17	0	∑*Power*_*i*_ ∙ *ON time*_*i*_ ∙ *elec*. *price*
Total costs (USD/d)	0.46	1.56	0.02	

## 4 Discussion

### 4.1 Mechanisms and selectivities of carbon removal and transformation

Batch testing with ozone, electrolysis and GAC demonstrated that each treatment was ultimately capable of removing significant portions of the DOC (≥95%). While the exposure times considered in these batch tests are not typical of one-pass treatment systems, this time frame is valid for water recycling systems where recalcitrant compounds will remain in the system, experiencing treatment over and over again. Comparing changes in DOC, AOC, absorbance, and size-distribution as a function of exposure can uncover reaction preferences.

Initial exposure of permeate to ozone demonstrated a net production of AOC. The DOC concentration was reduced, but the oxidation of a portion of the DOC transformed this DOC to AOC. Ozone also demonstrated a potential selectivity for reducing absorbance ~3–4 faster than DOC, and produced a shift in size-distribution to smaller compounds. These behaviors are consistent with the scientific understanding of ozone oxidation. Ozone is a highly selective oxidant, exhibiting preference for electron-rich moieties (Hoigné and Bader, 1983; Von Gunten, 2003). The carbon-carbon, carbon-nitrogen and carbon-oxygen double bonds, which contribute chromophore properties to compounds within the water (Gürses et al., 2016), are favorable reaction targets for ozone. The preference for ozone removing larger compounds (González et al., 2013; Zietzschmann et al., 2015), and the net formation of AOC have also been observed in previous studies (van der Kooij et al., 1989; Chen et al., 2018). The decomposition of ozone can lead to hydroxyl radical formation (Von Gunten, 2003), which has a higher oxidation potential than ozone (2.8 vs. 2.07 V). Our results suggest that selective ozone oxidation is dominant when compounds that can be oxidized by ozone are present, but less-selective hydroxyl radial oxidation ultimately mineralizes remaining ozone-resistant compounds.

Electrolysis treatment demonstrated a shorter period of net AOC production than ozonation and less preference for removal of absorbance without impacting the size-distribution. Though hydroxyl radicals can oxidize compounds that ozone cannot, hydroxyl radical maintains a reduced preference for more-easily-oxidized targets. The selective behavior observed with color, occurred at the very start of the batch experiment, and the portion of the total DOC associated with color may be very small. Our results demonstrate that this initial selective behavior for color-contributing compounds does not impact non-selective oxidation in size-distribution.

Batch testing with GAC did not demonstrate any selective behavior. The removal mechanism in our GAC batch test was limited to chemical and physical mechanisms because the use of virgin GAC and the short duration of the experiment (~3 h) limited significant bacterial inclusion. Other studies have indicated that the size relationship between GAC pores and target compounds in the water can have a significant impact on removal efficiency (Karanfil and Kilduff, 1999; Bhatnagar and Sillanpää, 2017). Our results could suggest that the largest compounds that pass through the biofilm on the BAMBi are also able to access the GAC pores, though we cannot conclusively determine that larger compounds were not removed through surface adsorption alone. The approximately neutral (pH ~ 8) of the water in our systems may have been lower than the ZPC (alkaline) of the GAC. This may have led to some degree of positive GAC surface charge, but it is difficult to understand the significance of this affect in a system that did not demonstrate removal preferences. A similar ability of GAC to effectively remove organic materials across the size range observed in our study (humics, building blocks, LMW organics and neutrals) has also been demonstrated previously in real and synthetic surface waters (Iriarte-Velasco et al., 2008; Velten et al., 2011).

When comparing the impacts of post-treatment at full-scale to the impacts in batch testing, each treatment was again effective at reducing DOC and absorbance. The inability of each post-treatment to impact the size distribution of carbon, can be attributed to the limited carbon content of the recycled water from the CWT (1 to 21 mg/L DOC) compared to the ~60 mg/L TOC contributed from the concentrated feed. The change from batch testing to full-scale testing did not change the oxidation preference of ozone or hydroxyl radicals, but it has limited the impacts that these preferences have on the fate of organic materials in the system.

### 4.2 Linking post-treatments and antibacterial performance in full-scale systems

The three antibacterial strategies that could contribute to reducing bacteria concentrations in our system are, (i) inhibiting growth by nutrient-limitation, (ii) inhibiting growth by oxidative stress, or (iii) inactivating bacteria though destructive oxidation. The concept of AOC-limitation as an antibacterial mechanism has been previously explored through the concept of biological stability. Biological stability describes an AOC threshold of approximately 0.01 mg/L in unchlorinated water or 0.1 mg/L in chlorinated water, below which the growth of bacteria is prevented or extremely limited (van der Kooij et al., 1989; Prest et al., 2016). The AOC concentrations observed at full-scale do not meet these thresholds indicating that AOC-limitation is not likely significant. The antibacterial performance is hence due to oxidation through ozone for ozone post-treatment, and through chlorine and other potential oxidation pathways for electrolysis post-treatment. In the GAC system, the absence of biological stability and the absence of antibacterial oxidation chemicals were consistent with the lack of improvement in bacteria concentrations. Removing organics from the system is still essential for an acceptable user experience, but active antibacterial strategies, as investigated in greater detail in our sister publication (Nguyen et al., 2017), dictate bacterial concentrations in this system.

### 4.3 Selecting an optimal process

Ozone and electrolysis treatments have each demonstrated antibacterial potency, but not without potential disadvantages and opportunities to improve on the implementations investigated in this study. The potential danger that ozone poses to users and to system components mandates effective safeguards and more-finely tuned dosing control than electrolysis or direct chlorination strategies. Though electrolysis costs were the highest, many aspects of electrolysis treatment are highly tunable including material, spacing, current density and flow characteristics, and there is potential to improve efficiency. The ability of electrolysis to produce chlorine, which can be both effective against regrowth and safe for users, offers tremendous advantages to the system that cannot be matched by ozone or GAC. This is particularly applicable in developing-world applications where the chlorine provides a longer-lasting antibacterial residual than ozone, and GAC does not provide any antibacterial chemical residual. The addition of GAC demonstrated significant benefits (DOC, AOC and color removal), without any evident disadvantages, at a cost that is relatively insignificant.

While GAC alone does not eliminate regrowth potential, adding electrolysis treatment after GAC would enable chlorine production to provide an antimicrobial residual. The significant removals in DOC and AOC achieved by the GAC would reduce the oxidation demand (ozone or chlorine) of the water, allowing a significant minimization of ozone or electrolysis treatment. If the ability of the electrolysis to produce hydroxyl radicals is not necessary, a simplified electrolysis unit could be optimized for chlorine production or chlorine could be dosed directly. While this study has sought to understand selectivities for removals and transformations of organic carbon, our analysis indicates that carbon selectivity is not ultimately linked to performance. Our process design recommendations are based on the stability and cost-efficiency of GAC to remove carbons of all sizes and the ability of ozone or electrolysis to reduce bacteria concentrations following treatment. The lifetime and potential biological removal contributions of the GAC need to be investigated further, and both ozone and electrolysis configurations need to be optimized, but pairing the BAMBi with hybrid post-treatment is a very promising strategy to deliver safe water for small-scale water recycling applications.

As we improve our technical understanding of what small-scale water recycling systems can achieve, we must also understand where these systems are needed and what constraints will be placed upon them. A decentralized hand washing water recycling system can have the greatest health impact in a challenging environment, perhaps with limited financial resources, limited control over how the system is used and limited technical support in the event of a breakdown. It is then essential to understand how treatment systems might perform under stressful conditions, but also what performance objectives (DOC, AOC, TCC, pathogens of concern) will quantitatively benefit users. This paper has step to demonstrate sufficient reduction and transformation of organic carbon to remove the perception of “dirtiness” of the water and to reduce the organic scavenging of antimicrobial oxidation chemicals (chlorine and ozone). There are more steps to follow, but we are going in the right direction.

## 5 Conclusions

Tis study closes a significant process knowledge gap in our understanding of how ozone, electrolysis and GAC post-treatments impact organic material in biological greywater recycling, and how these interactions affect overall system objectives. Our results did indicate that a biological water treatment system, paired with the post-treatment technology examined in this study can produce water to a standard that is perceptibly acceptable to the user, while containing comparable bacteria concentrations as municipal drinking water. Achieving this performance was not clearly driven by reaction selectivities of the examined post-treatment technologies. The inability of any of our systems to reduce AOC concentrations to the threshold of biological stability indicated that this may not be a realistic antibacterial strategy for similar decentralized water recycling systems. We instead observed the value and recommend the inclusion of active antibacterial chemicals. We further recommend employing hybrid post-treatment systems with individual components specialized for carbon removal (GAC) and antibacterial performance (ozone or electrolysis) respectively.

## CRediT authorship contribution statement

**Christopher Ziemba:** Conceptualization, Investigation, Data curation, Formal analysis, Visualization, Writing - original draft. **Odile Larivé:** Investigation, Writing - review & editing. **Eva Reynaert:** Formal analysis, Visualization, Writing - review & editing. **Theo Huisman:** Investigation, Writing - review & editing. **Eberhard Morgenroth:** Conceptualization, Supervision, Writing - review & editing.

## Declaration of competing interest

The authors declare that they have no known competing financial interests or personal relationships that could have appeared to influence the work reported in this paper.

## Appendix A Supplementary data

Supplementary data Image 1
