# Supplementary material for: Linking transformations of organic carbon to post-treatment performance in a biological water recycling system
Source: Sci Total Environ. 2020 Jun 15;721:137489. doi: 10.1016/j.scitotenv.2020.137489 (PMC7237881; doi:10.1016/j.scitotenv.2020.137489)
Supplement: Supplementary file 1 — Supplementary data [file mmc1.pdf]

Transformations of organic carbon during ozone, electrolysis and GAC post-treatments in a biological water recycling system

Supporting Information

Christopher Ziemba [a, b], Odile Larivé [a, c], Eva Reynaert [a, b],

Theo Huisman [a, b, d], and Eberhard Morgenroth [a, b, *]

[a] Eawag: Swiss Federal Institute of Aquatic Science and Technology, 8600 Dübendorf, Switzerland

[b] ETH Zürich, Institute of Environmental Engineering, 8093 Zürich, Switzerland

[c] (current address) Ecole Polytechnique Fédérale de Lausanne (EPFL) Lausanne, Environmental Chemistry Laboratory, 1015 Lausanne, Switzerland

[d] (current address) King Abdullah University of Science and Technology (KAUST), Thuwal 23955-6900, Saudi Arabia

* Corresponding author. E-mail address: Eberhard.Morgenroth@eawag.ch

The supporting information contains the following:

Section S.1. describes how this study is based in the Blue Diversion Autarky Toilet project and explains how the wash water composition has been based on experience field testing this prototype source-separating toilet, and how it is recreated in the laboratory. This SI section provides background for the study broadly, but also specifically for the introduction of the feed.

Section S.2. provides additional detail about the start-up and operation of the BAMBi.

Section S.3. provides additional detail about Liquid chromatography with organic carbon detector (LC-OCD) and dissolved organic carbon (DOC) measurements.

Section S.4. provides expanded details on assimilable organic carbon (AOC), total cell count (TCC) and intact cell count (ICC) processing.

Section S.5. provides additional data collected during batch testing

Section S.6. provides additional data collected during full-scale testing

Section S.7. details the methods and justifications for estimating investment and operating costs for each post-treatment implemented at full-scale.

Section S.8. provides photo of Water Wall during previous field tests.

Section S.1. Blue Diversion Autarky Toilet wash water context and composition

The BAMBi systems utilized in this study have been originally designed to serve the Blue Diversion Toilet (BDT) as part of the Bill and Melinda Gates Foundation Reinvent the Toilet Challenge. The BDT is a source separating toilet designed to provide safe sanitation to urban parts of the world that may have limited access to safe water sources and have no access to sewer facilities (Larsen et al. 2015). The toilet was designed to create one waste stream of urine and one waste stream of feces, so resources can be recovered from each waste. The original version of the BDT featured collection and centralized treatment of the urine and feces, while the updated version, called the Blue Diversion Autarky Toilet (BDAT), includes integrated urine and feces treatment. Both versions provide a water recycling component to supply safe water for hand washing, anal cleansing and flushing out the bowl where urine and washing water is collected. Though the BDT is a source separating toilet, field testing has demonstrated that 1-2% of the urine and feces produced by users are able to enter the water recycling system.

The wash water utilized in this study is formulated from actual human waste and soap to recreate what was observed in field testing. Human feces and urine were collected at Eawag (Dübendorf, Switzerland). Bags of feces were frozen, diluted 100% in tap water and homogenized in a blender before feeding. Urine was added to the feeding tank after up to 24 h storage at room temperature. Soap was prepared from components: 140 g/L sodium dodecyl sulfate (VWR, Radnor, Pennsylvania, USA), 50 g/L glycerol (Merck, Darmstadt, Germany), 10 g/L NaCl (Merck, Darmstadt, Germany), and 0.72 g/L lactic acid (Alfa Aesar, Ward Hill, Massachusetts, USA) in tap water. The chemical characteristics of the concentrated feed water were approximately 1200 mg/L TOC, 300 mg/L total N, 100 mg/L-N combined ammonium and ammonia, and 200 mg/L chloride. The daily loading of carbon and nutrients divided by the daily volume of water processed in the BDT would create a wash water strength similar to that of greywater.

Section S.2. Additional details of BAMBi operation

Figure S1 depicts the 3 BAMBi units operated in this study, their feeding and waste plumbing and how post-treatments were integrated into each system. Coarse bubble (~3 mm diameter) aeration was

introduced directly below the membrane module at a rate of 3 L/min. The daily feeding of 3 L/day was divided in a series of 40 events. Each event consisted of 75 mL of the concentrated feed and 1425 mL of water from the CWT being pumped back to the BAMBi. Treated water was removed from the CWT for each event to maintain a constant system volume. The combined 1.5 L of wash water added to the BAMBi for each event represents the combined inputs from hand washing, anal/personal cleansing and washing out the toilet bowl, as produced in the Blue Diversion Autarky Toilet. The 40 events correspond to 40 usages of the toilet, and the distribution of events throughout the day was based on expected usage, with high frequency at breakfast, lunch and dinner. The activation of the pumps controlling the concentrated feed, permeate, waste and the return of water from the CWT to the BAMBi was managed using process control hardware (Endress + Hauser AG Reinach BL, Switzerland), and automation softwares Codesys (3S-Smart Software Solutions GmbH, Kempten, Germany) and CitectSCADA (Schneider Electric, Rueil-Malmaison, France). Each BAMBi was initially started with 50 L of tap water, 0.5 L of activated sludge (from a conventional wastewater treatment plant in Switzerland) and 0.5 L of sludge from a urine nitrification reactor (Fumasoli et al., 2016). BAMBis were then operated for 2 months to allow the bacteria to acclimate and performance to stabilize before the more intensive data collection for this study began. No fouling control was performed on the membrane during testing. Approximately 2.75 L/d of CWT water was wasted to maintain system volume (~0.25 L/d lost to evaporation).

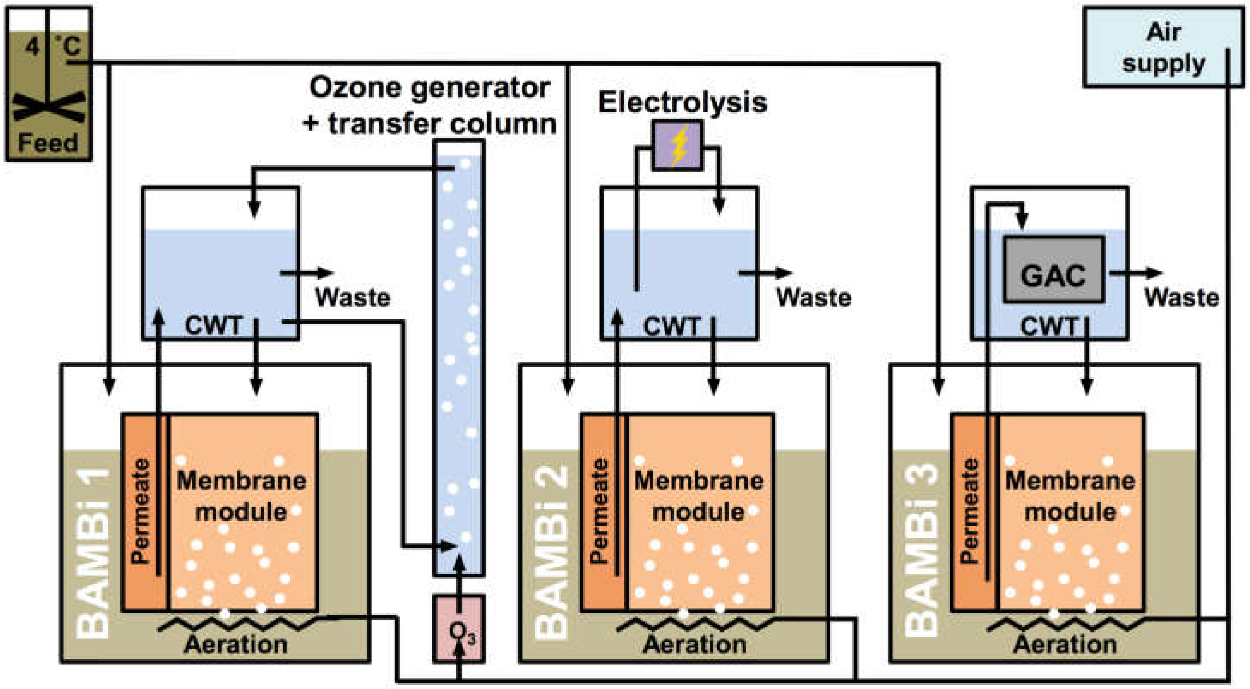

Figure S1. Schematic of 3 biologically activated membrane bioreactor (BAMBi) systems. CWT: clean water tank. GAC: granular activated carbon.

Section S.3. Details of LC-OCD and DOC measurements

The separation range for the LC-OCD column was with a range of 100–20,000 Da, and the lower quantification limit for DOC testing on the LC-OCD was 50 µg/L. All glassware for LC-OCD sampling and analysis was heated in a clean furnace to a temperature of 450 ˚C and held at this temperature for 12 h to incinerate any trace carbon residue. All samples for LC-OCD and DOC testing were each spiked with of 46 g/L sodium thiosulfate solution (0.25 mL per 50 mL of sample) to quench any oxidants.

Section S.4. Details of AOC, TCC and ICC processing

Samples for AOC processing were collected using 50 mL syringes and first filtered, with prewashed (50 mL of deionized water) 0.2 µm polyethersulfone filters (Pall Port Washington, New York, USA), into 60 mL glass vials and 1 mL of inoculum was added. Mixed samples were divided into three 45 mL glass vials, capped with PTFE-lined lids, incubated for 3 days at 30 ˚C without light and measured for TCC. AOC was

calculated using the equation 1 µg AOC = 10^7 cells (Hammes et al. 2005). Inoculum was prepared from equal parts of (i) Evian water (Évian-les-Bains, France), (ii) permeate water from a BAMBi system fed the same as in this study, (iii) permeate water from a BAMBi system fed a synthetic greywater and (iv) water collected from the Chriesbach stream (Dübendorf, Switzerland). The BAMBi waters and the stream water were twice centrifuge washed (10 min at 3500 g) and re-suspended in mineral buffer (LeChevallier et al. 1993). The TCC of the inoculum was approximately 100 cells/mL. All glassware was muffled at 450 ˚C for 12 hours. All TCC, ICC and AOC samples were spiked with 0.25 mL of 46 g/L sodium thiosulfate solution per 50 mL of sample. The lower limit of quantification for TCC and ICC was 970 cells/mL and the lower limit for AOC quantification was 2.1 µg/L.

Section S.5. Additional data for batch testing

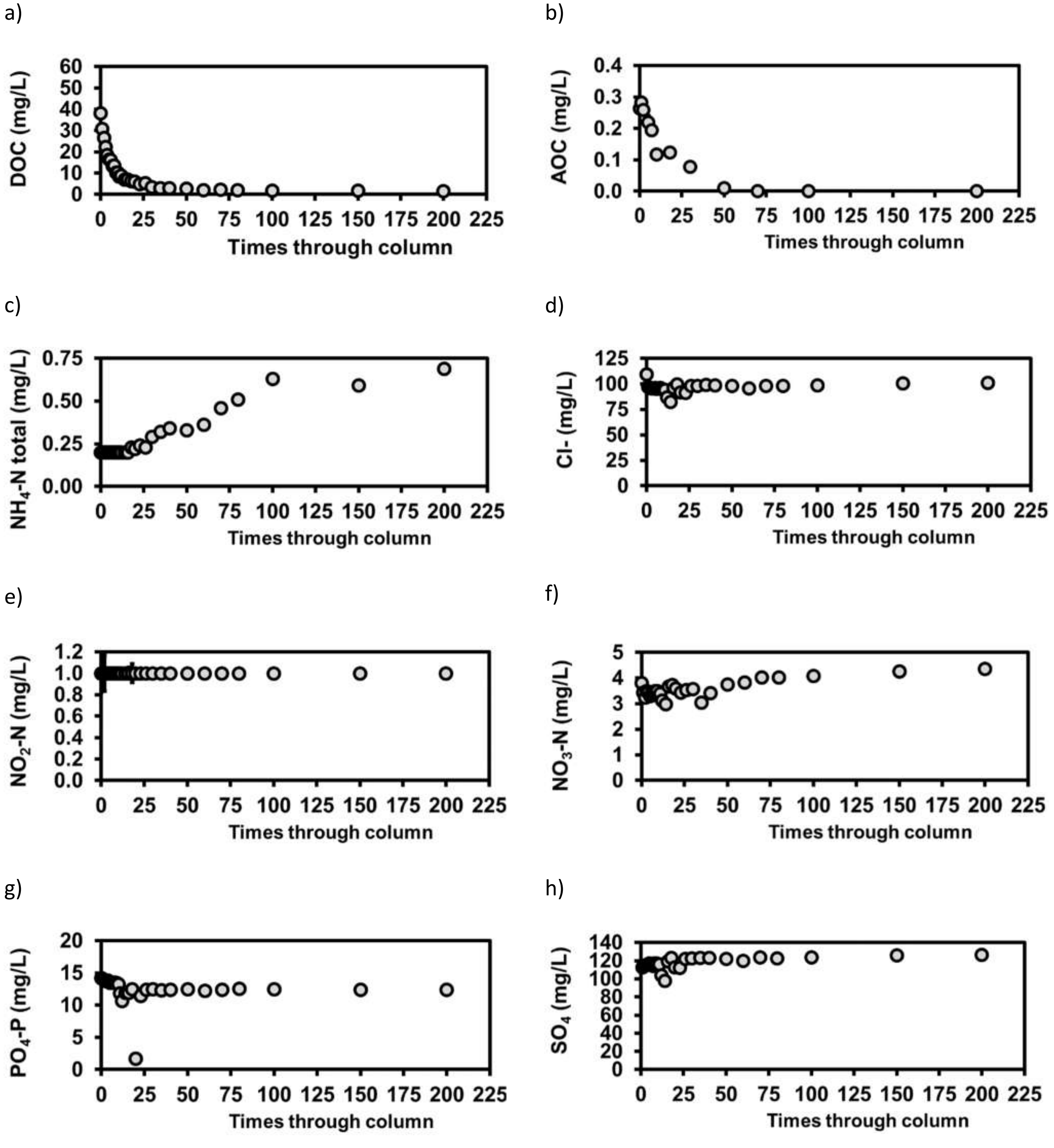

Figure S2. GAC batch testing. NH_4-total indicates that these data represent the combined values for ammonia and ammonium species.

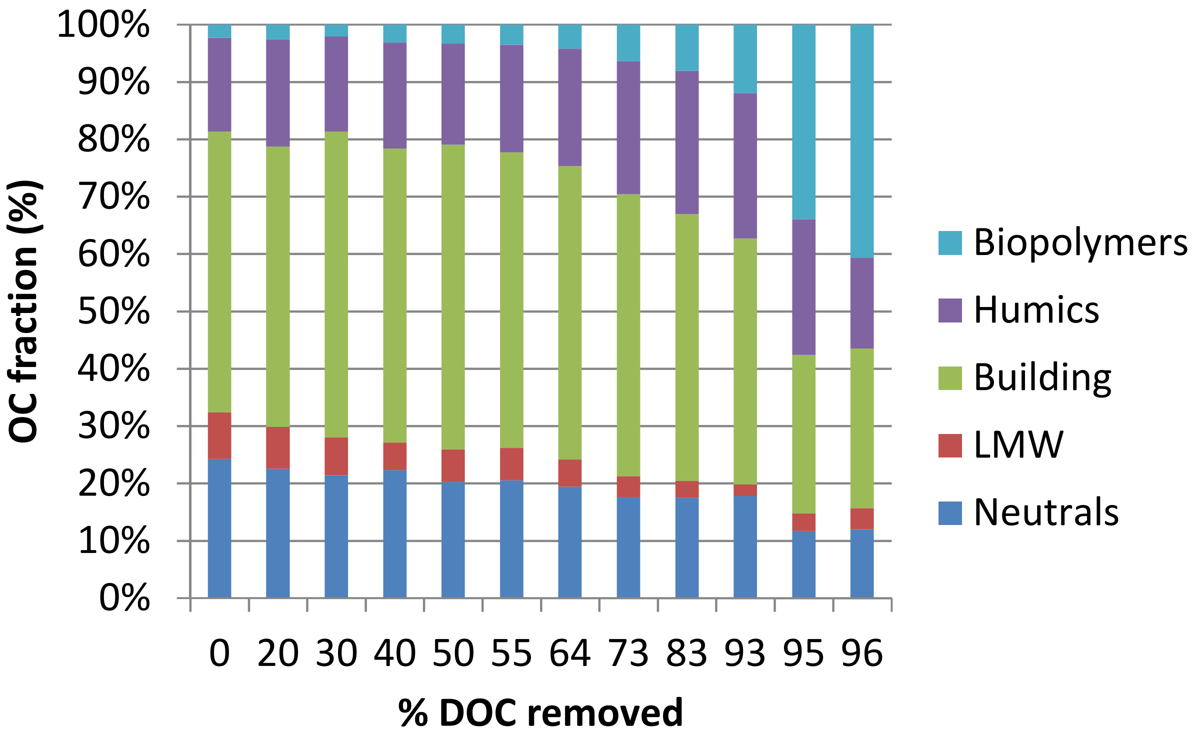

Figure S3. Size-distribution profiles from GAC batch testing. Each sample is identified by the corresponding percentage of DOC removed by the treatment at the time of sample collection. This figure includes a peak that occurs at the same elution time as the biopolymer calibration peak, however this peak has not been included in the main text because we believe this peak may be contamination.

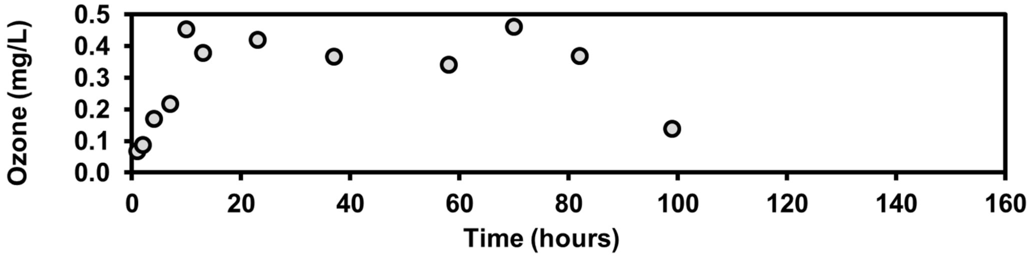

Figure S4. Ozone measured in ozone batch test.

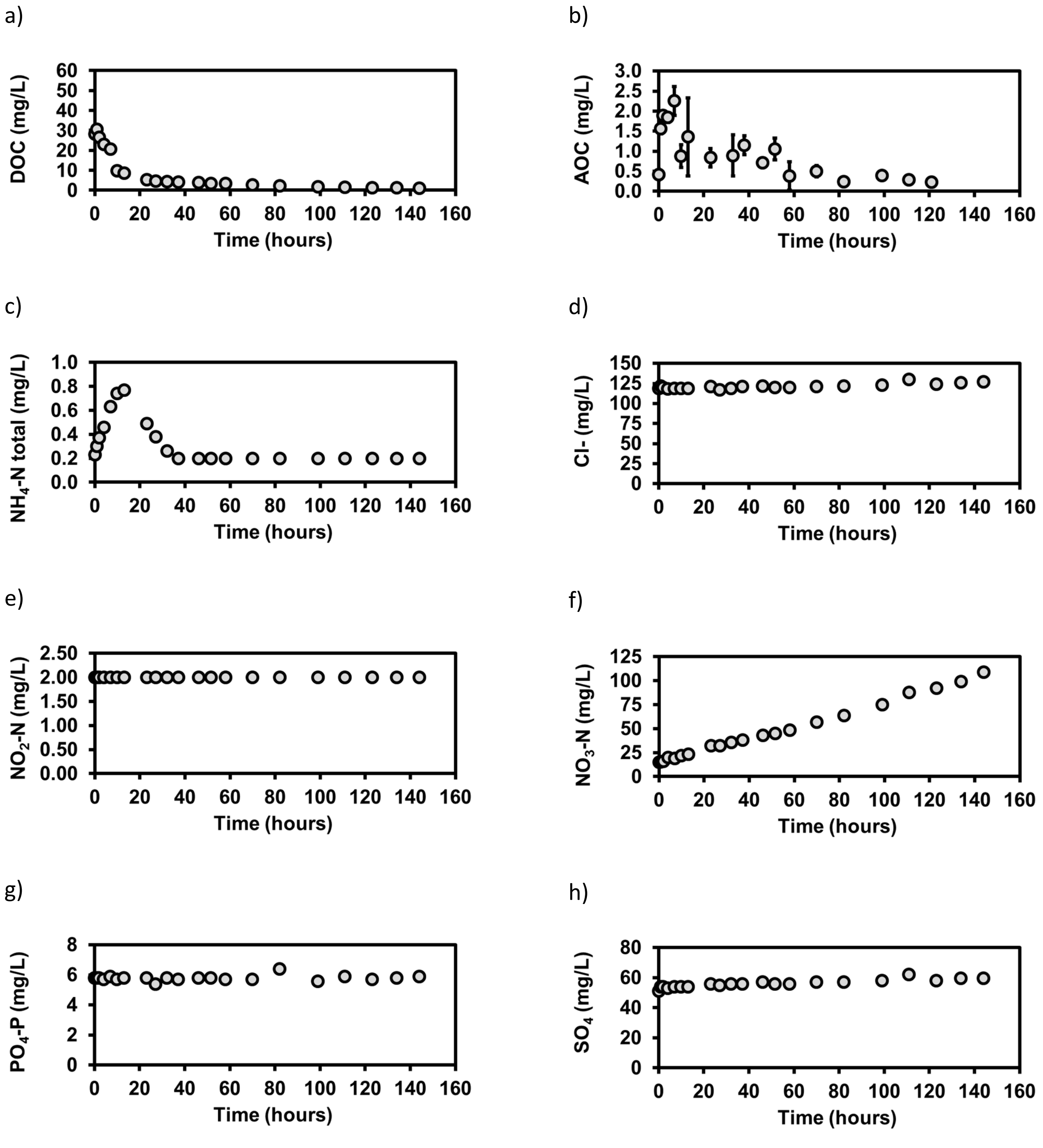

Figure S5. Ozone batch testing. NH_4-total indicates that these data represent the combined values for ammonia and ammonium species.

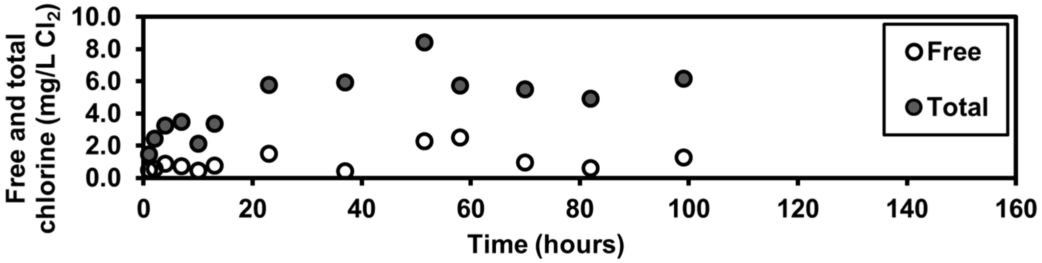

Figure S6. Free and total chlorine measured in electrolysis batch test. Open circles represent free chlorine and closed circles represent total chlorine.

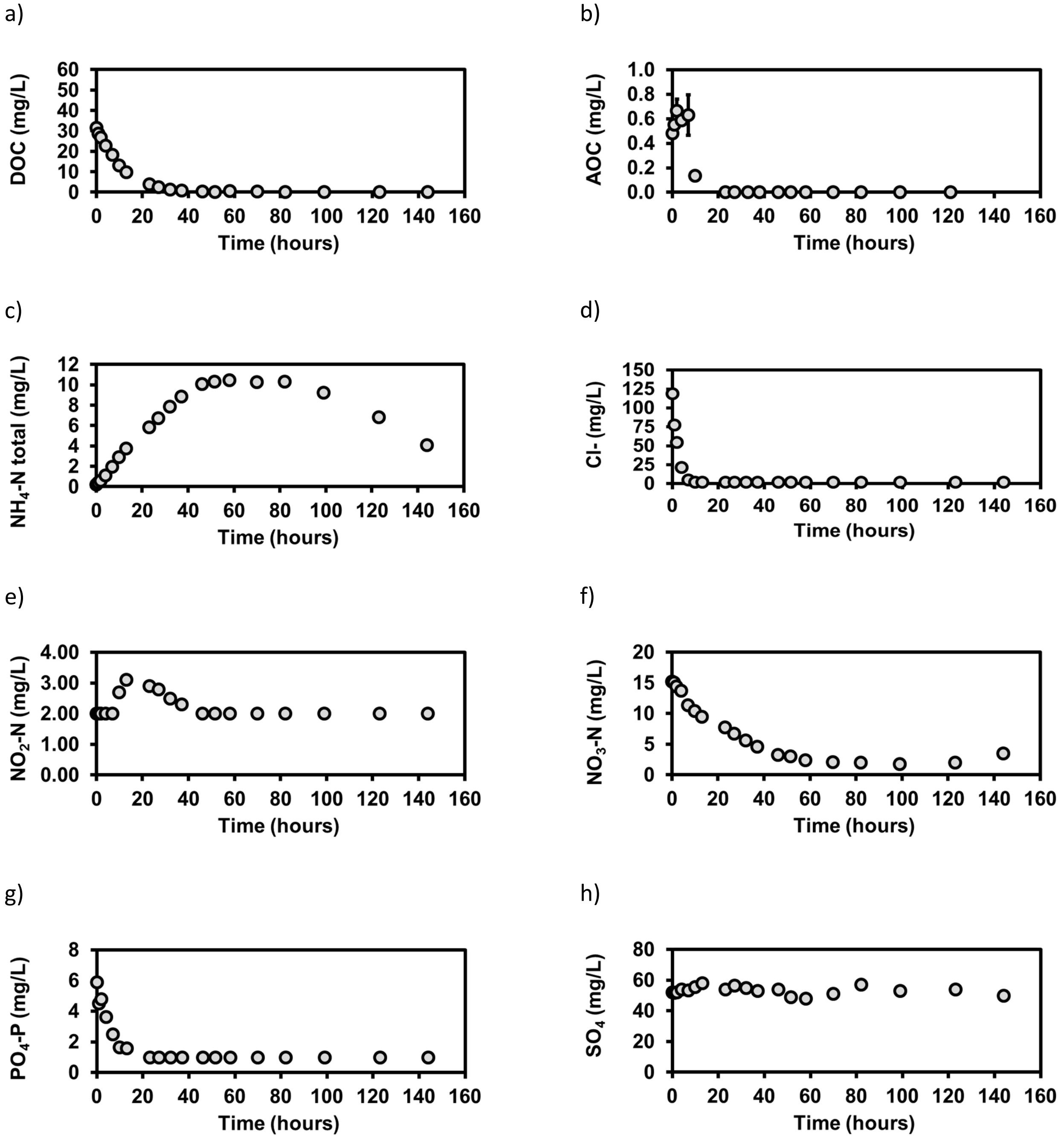

Figure S7. Electrolysis batch testing. NH_4-total indicates that these data represent the combined values for ammonia and ammonium species.

Section S6. Additional data for full-scale testing

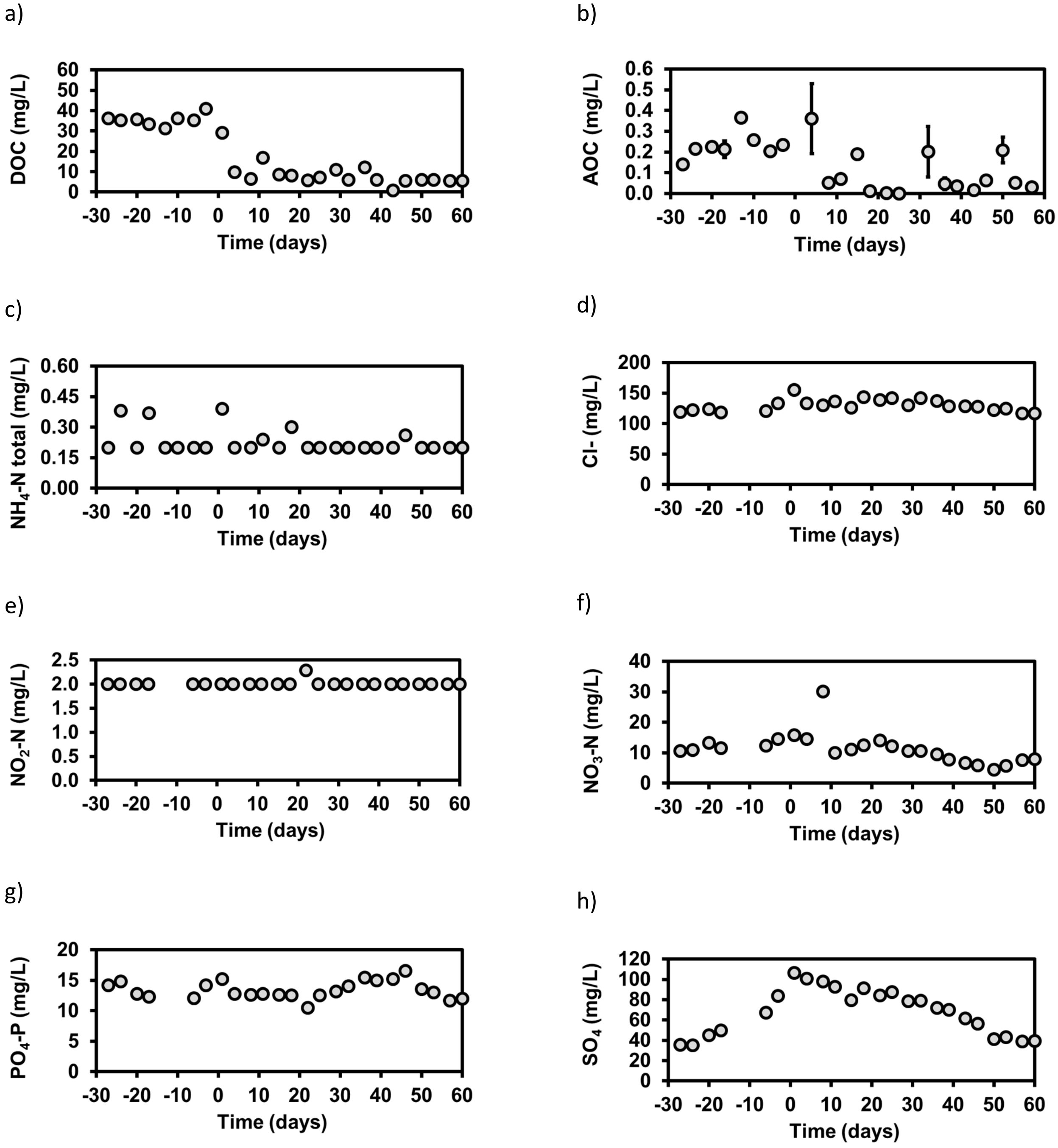

Figure S8. Full-scale operation in CWT with ozone treatment added at day zero. NH_4-total indicates that these data represent the combined values for ammonia and ammonium species.

a)

b)

c)

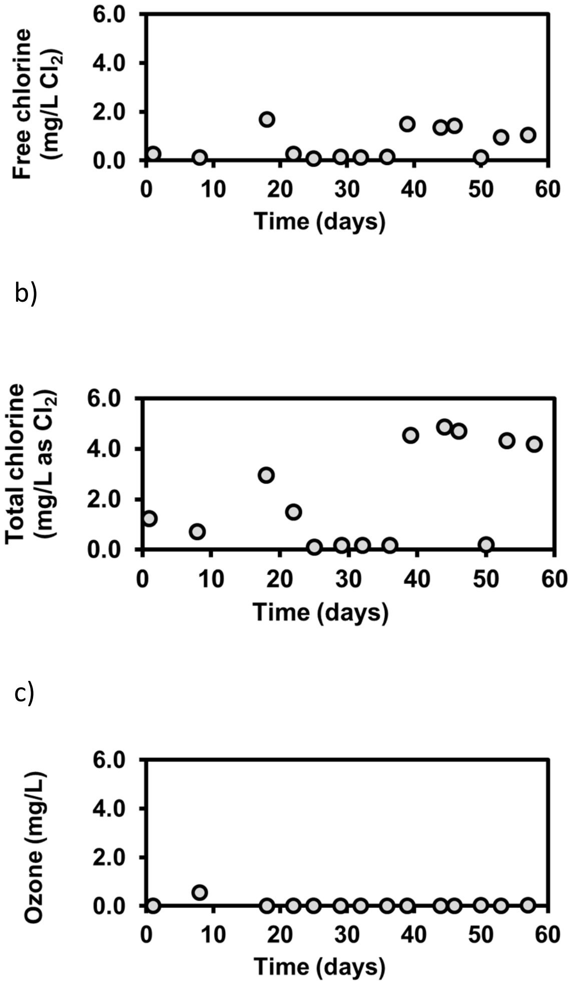

Figure S9. Free chlorine (a) and total chlorine (b) measured in the CWT during full-scale testing with electrolysis, and ozone (c) measured in the CWT during full-scale ozone testing.

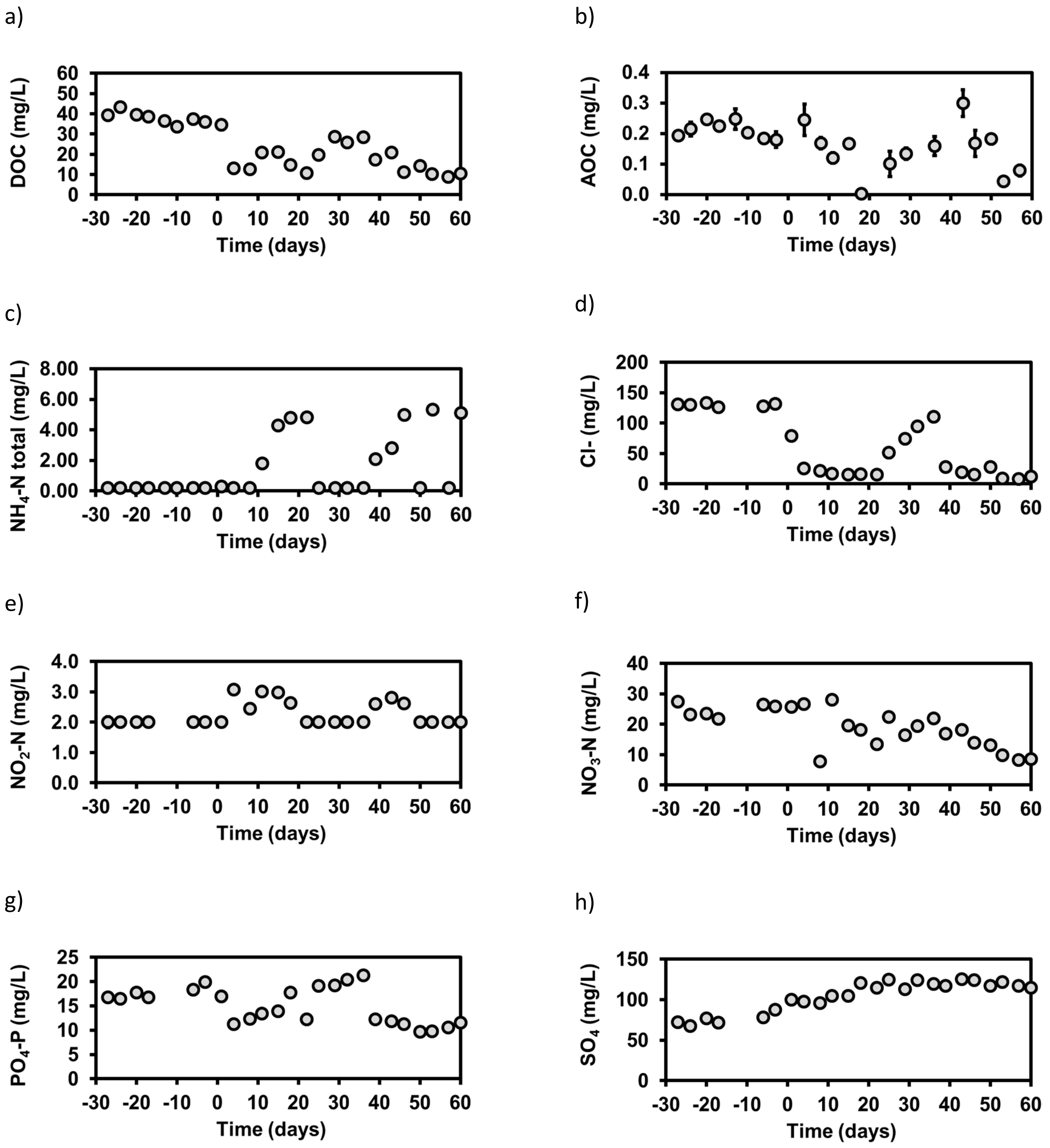

Figure S10. Full-scale operation in CWT with electrolysis treatment added at day zero. NH_4-total indicates that these data represent the combined values for ammonia and ammonium species.

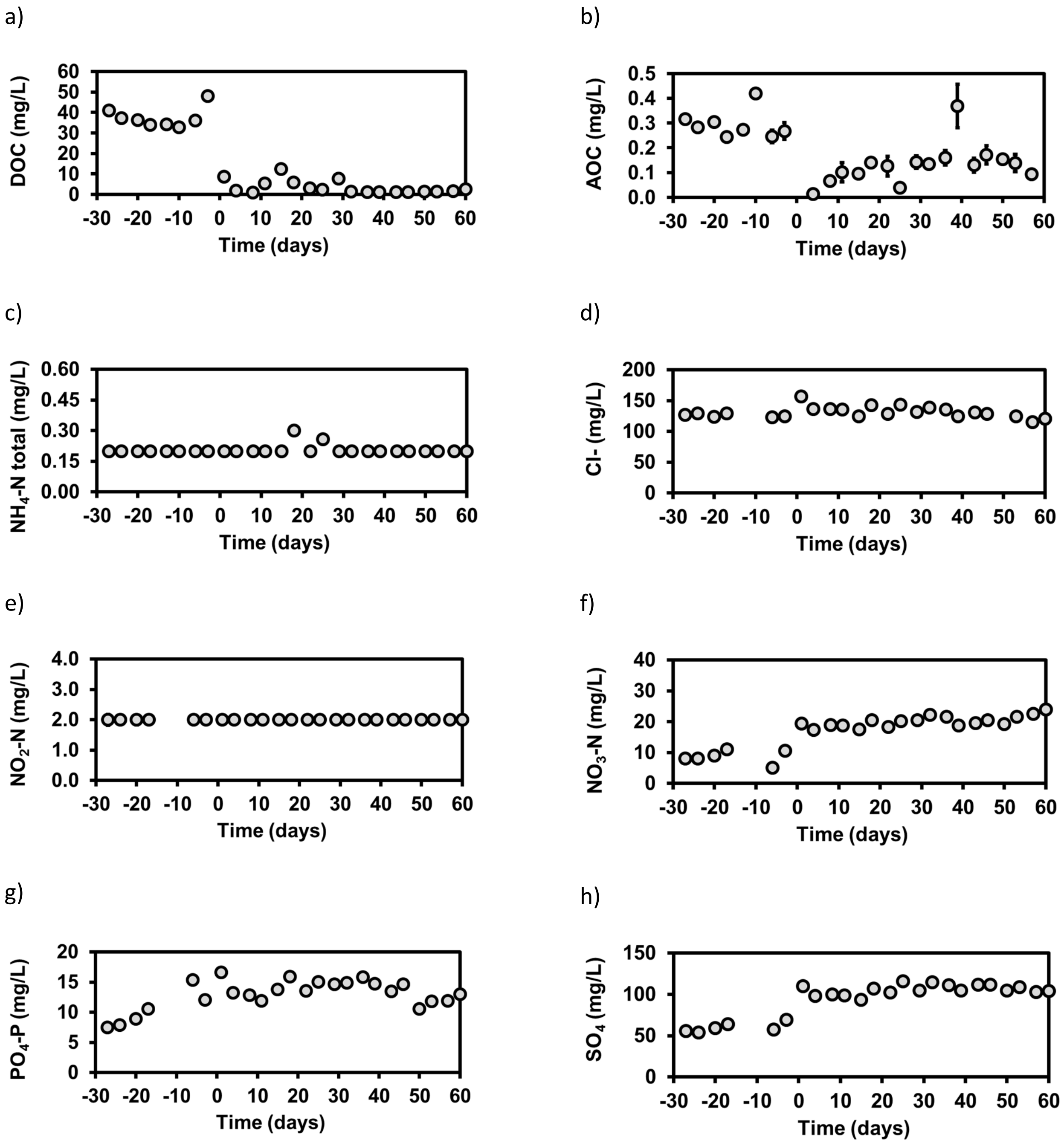

Figure S11. Full-scale operation in CWT with GAC treatment added at day zero. NH_4-total indicates that these data represent the combined values for ammonia and ammonium species.

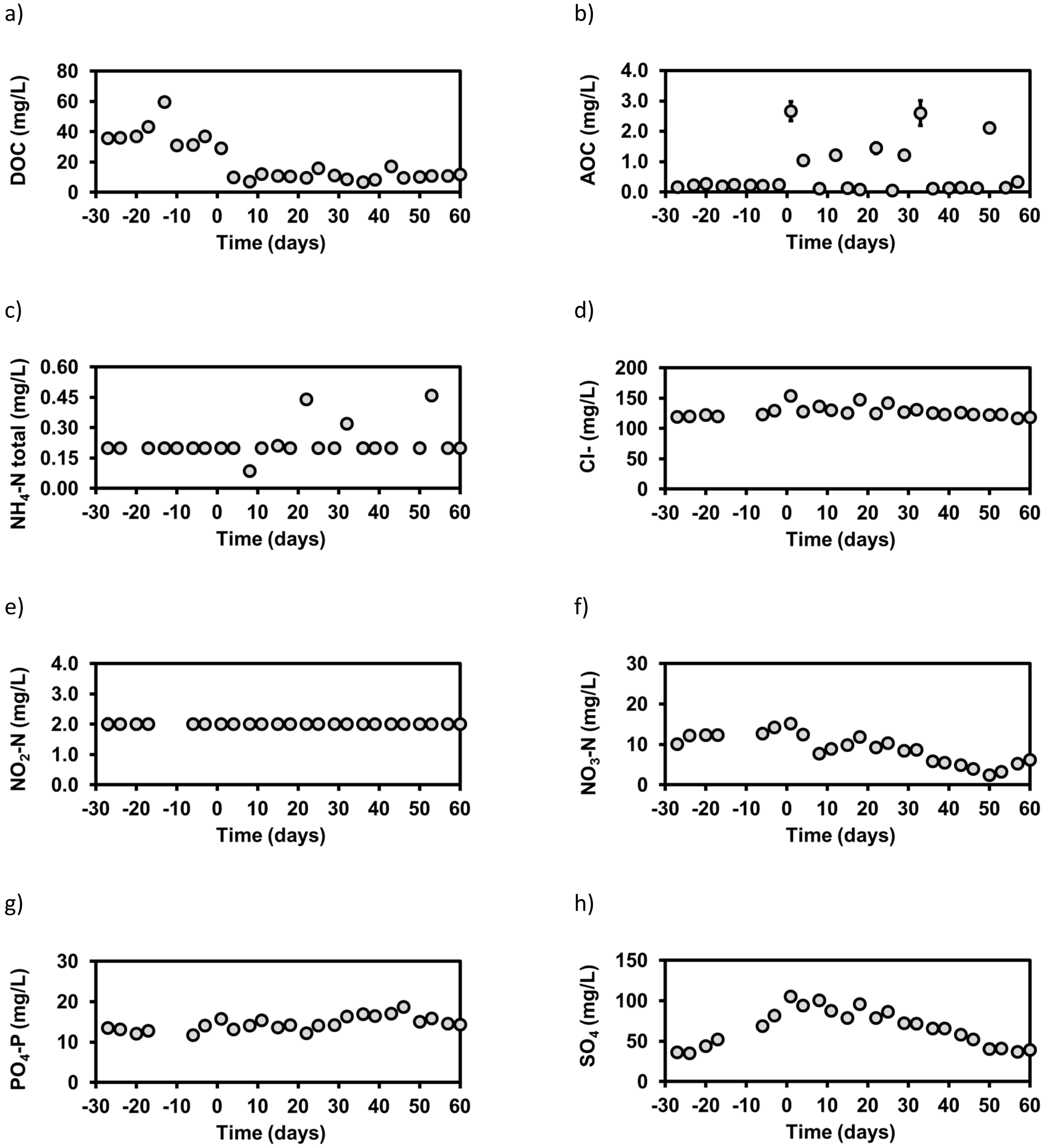

Figure S12. Full-scale operation in permeate with ozone treatment added at day zero. NH_4-total indicates that these data represent the combined values for ammonia and ammonium species.

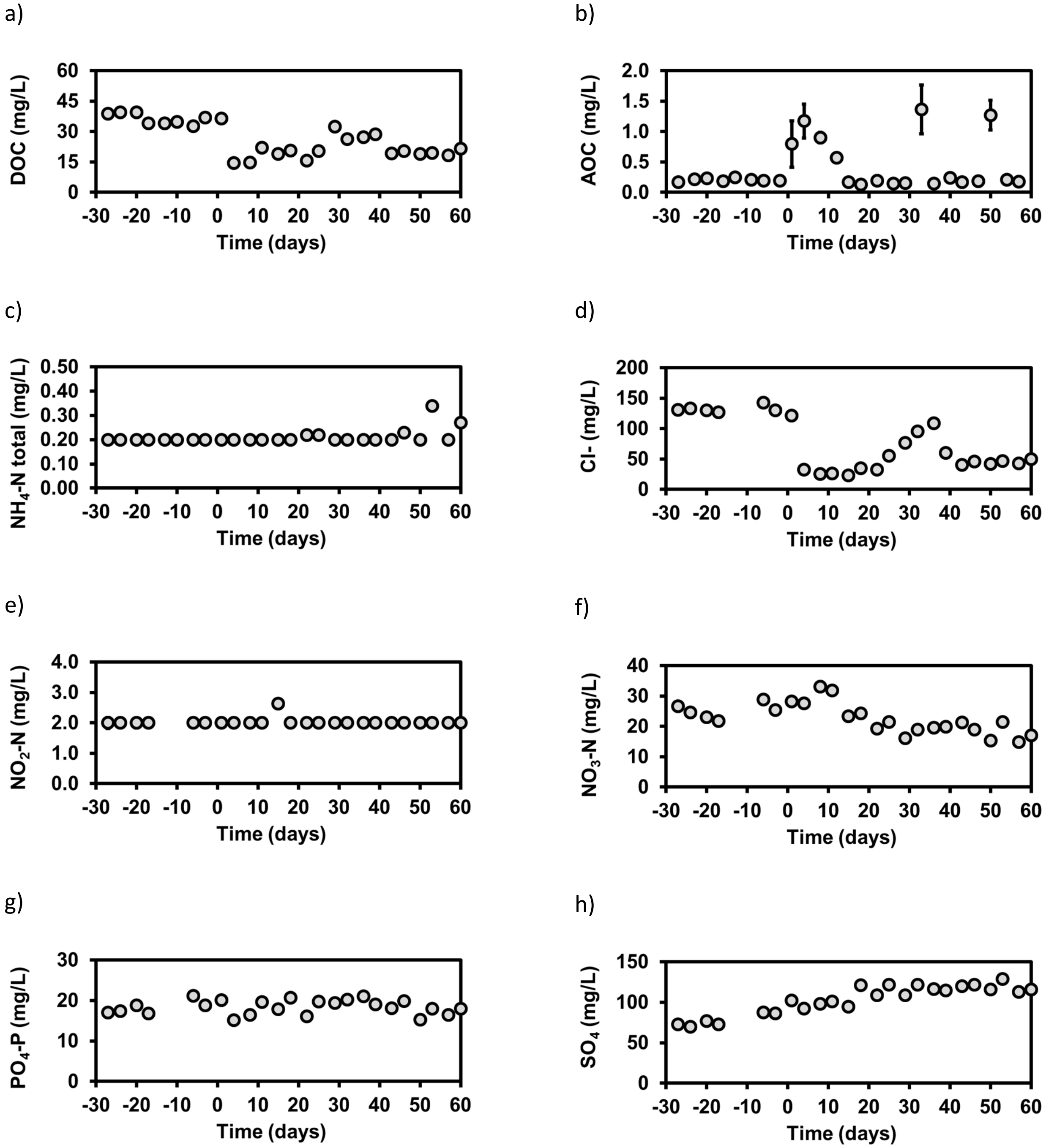

Figure S13. Full-scale operation in permeate with electrolysis treatment added at day zero. NH_4-total indicates that these data represent the combined values for ammonia and ammonium species.

a)

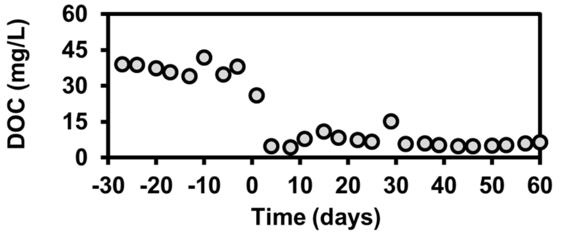
DOC (mg/L)
60
45
30
15
0
-30 -20 -10 0 10 20 30 40 50 60
Time (days)

c)

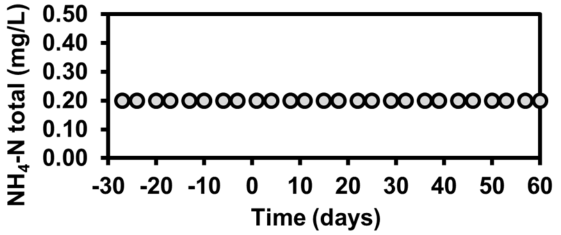
NH_4-N total (mg/L)
0.50
0.40
0.30
0.20
0.10
0.00
-30 -20 -10 0 10 20 30 40 50 60
Time (days)

e)

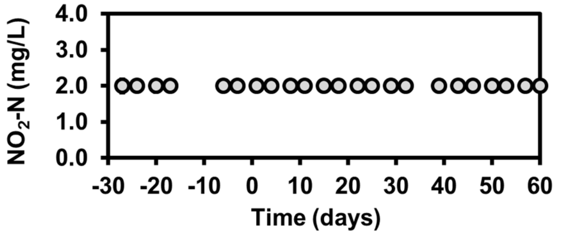
NO_2-N (mg/L)
4.0
3.0
2.0
1.0
0.0
-30 -20 -10 0 10 20 30 40 50 60
Time (days)

g)

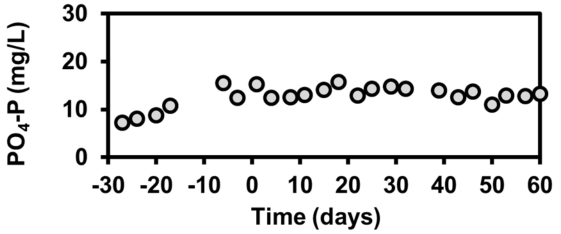
PO_4-P (mg/L)
30
20
10
0
-30 -20 -10 0 10 20 30 40 50 60
Time (days)

b)

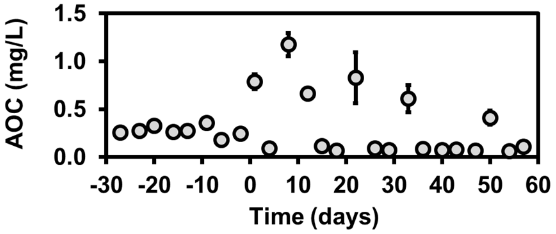
AOC (mg/L)
1.5
1.0
0.5
0.0
-30 -20 -10 0 10 20 30 40 50 60
Time (days)

d)

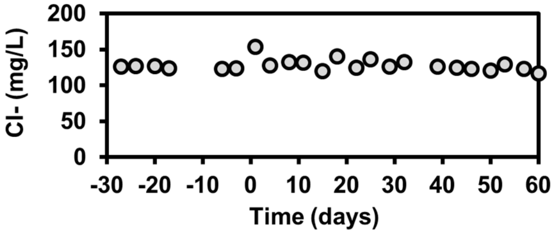
Cl- (mg/L)
200
150
100
50
0
-30 -20 -10 0 10 20 30 40 50 60
Time (days)

f)

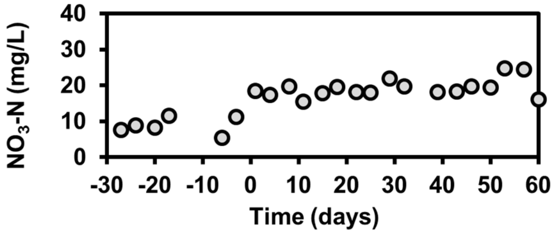
NO_3-N (mg/L)
40
30
20
10
0
-30 -20 -10 0 10 20 30 40 50 60
Time (days)

h)

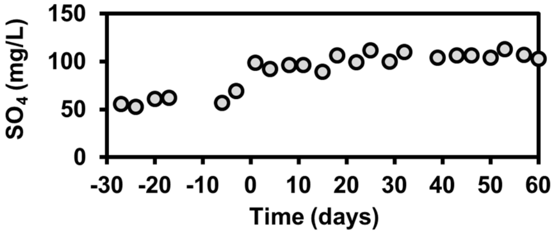
SO_4 (mg/L)
150
100
50
0
-30 -20 -10 0 10 20 30 40 50 60
Time (days)

Figure S14. Full-scale operation in permeate with GAC treatment added at day zero. NH_4-total indicates that these data represent the combined values for ammonia and ammonium species.

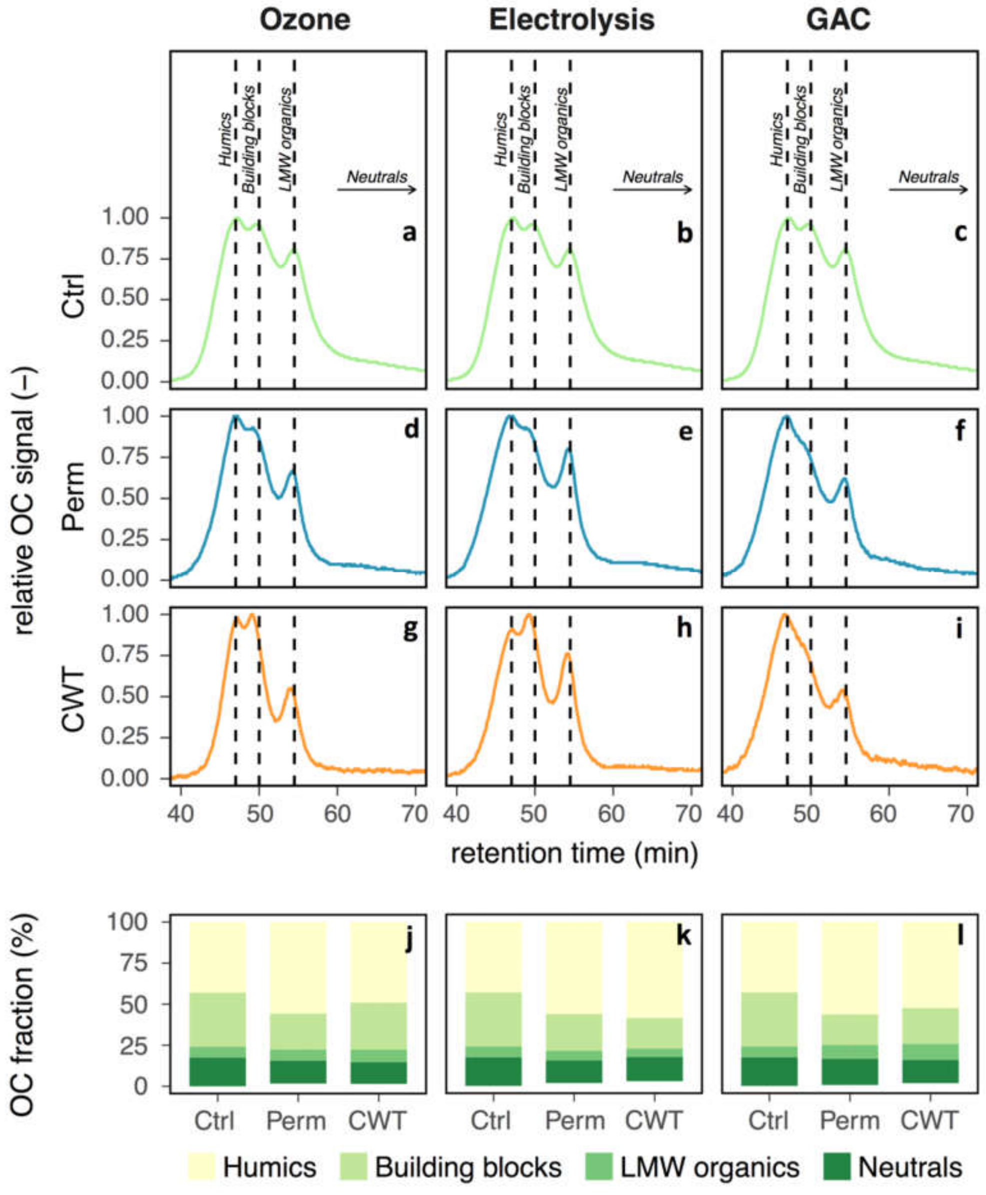

Figure S15. LC-OCD distribution profiles for BAMBi permeate before addition of post-treatment (parts a-c), permeate with post-treatment installed (parts d-f), and water in the CWT with post-

treatments installed (parts g-i). All LC-OCD distributions are scaled to have maximum signal values of 1. Parts j-l illustrate relative fraction distributions. OC: organic carbon, CWT: clean water tank, Ctrl: control (no post-treatment).

Section S.7. Detailed estimation investment and operating costs

These estimates utilize the same ozone generator and electrolysis unit as in batch and full-scale testing. Ozone is modeled as operating continuously through the day using the same ozone generator as in experimental testing with an estimated lifespan of 3 years. Aeration for ozone production is modeled as an EHEIM air pump (model 200, EHEIM, Deizisau, Germany) and recirculation with a submersible pump (Renkforce model number 539090-FH, Barwig Wasserversorgung, Bad Karlshafen, Germany). Electrolysis is modeled with the same electrolysis module used in experimental testing, operating at 2.5 A, 12.2 V and an on/off cycle of 5 min on to 1 min off. The lifespan of 800 days is estimated by the manufacturer based on the expected duty in kAh. Electrolysis utilizes the same recirculation pump as ozone. GAC treatment utilizes a volume of 6 L, with an estimated cost of 2 USD/kg and an estimated lifetime of 1 year. Energy costs were calculated based on average electricity prices in Zurich, Switzerland.

Section S.8. Photo of Water Wall during previous field-testing in Nairobi in 2014 (by Rahel Künzle).

The Water Wall in the current study is similar to the photo below but it was operated with simulated addition of small amounts of urine and feces.

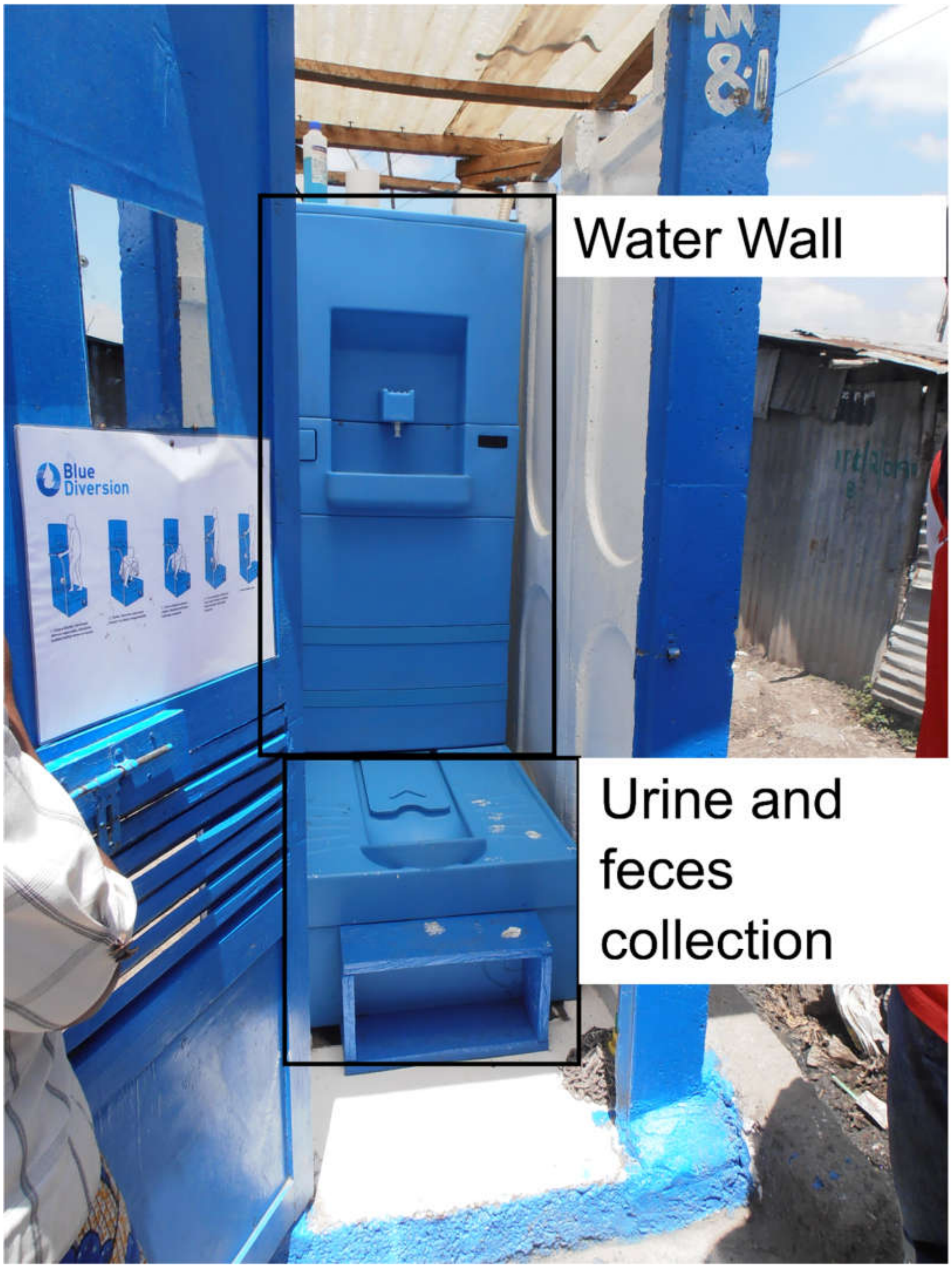

References:

Larsen, T.A., Gebauer, H., Gründl, H., Künzle, R., Lüthi, C., Messmer, U., Morgenroth, E., Niwagaba, C.B. and Ranner, B., Blue diversion: A new approach to sanitation in informal settlements. *Journal of Water Sanitation and Hygiene for Development* **2015,** 5(1), 64-71.

LeChevallier, M.W., Shaw, N.E., Kaplan, L.A. and Bott, T.L., 1993. Development of a rapid assimilable organic carbon method for water. *Applied and Environmental Microbiology* **1993,** 59(5), 1526-1531.

Hammes, F. A.; Egli, T., New method for assimilable organic carbon determination using flow-cytometric enumeration and a natural microbial consortium as inoculum. *Environmental Science and Technology* **2005,** 39, (9), 3289-3294.
